# PET/CT Imaging in Treatment Planning and Surveillance of Sinonasal Neoplasms

**DOI:** 10.3390/cancers15153759

**Published:** 2023-07-25

**Authors:** Sinan Akay, Janet H. Pollard, Assim Saad Eddin, Aiah Alatoum, Sedat Kandemirli, Ali Gholamrezanezhad, Yusuf Menda, Michael M. Graham, Ahmad Shariftabrizi

**Affiliations:** 1 Division of Nuclear Medicine, Department of Radiology, Carver College of Medicine, University of Iowa, Iowa City, IA 52242, USA; 2 Department of Radiology, Keck School of Medicine, University of Southern California (USC), Los Angeles, CA 90030, USA

**Keywords:** sinonasal malignancy, PET/CT, FDG, somatostatin analogs

## Abstract

**Simple Summary:**

Sinonasal cancers are rare types of cancer that are often detected at a late stage, making them difficult to treat. To monitor these cancers closely, advanced imaging techniques are used. One such technique is called “Whole-body ^18^F-FDG PET/CT”, which is an excellent tool in detecting and staging these cancers. For certain types of tumors, other imaging tracers like ^11^C-choline and ^68^Ga-DOTATATE/DOTATOC are helpful for planning treatment and monitoring progress. In this review, we talk about how PET/CT can aid in the diagnosing, determining the extent of the disease, treatment planning, and evaluating the treatment response for different types of sinonasal tumors. The review also discusses some newer radiotracers used for PET scanning, that are proving useful in diagnosing and staging sinonasal cancers.

**Abstract:**

Sinonasal cancers are uncommon malignancies with a generally unfavorable prognosis, often presenting at an advanced stage. Their high rate of recurrence supports close imaging surveillance and the utilization of functional imaging techniques. Whole-body ^18^F-FDG PET/CT has very high sensitivity for the diagnosis of sinonasal malignancies and can also be used as a “metabolic biopsy” in the characterization of some of the more common subgroups of these tumors, though due to overlap in uptake, histological confirmation is still needed. For certain tumor types, radiotracers, such as ^11^C-choline, and radiolabeled somatostatin analogs, including ^68^Ga-DOTATATE/DOTATOC, have proven useful in treatment planning and surveillance. Although serial scans for posttreatment surveillance allow the detection of subclinical lesions, the optimal schedule and efficacy in terms of survival are yet to be determined. Pitfalls of ^18^F-FDG, such as post-surgical and post-radiotherapy crusting and inflammation, may cause false-positive hypermetabolism in the absence of relapse.

## 1. Introduction

Sinonasal malignancies are rare diseases, accounting for 3% to 5% of all head and neck malignant neoplasms [1]. They contain several histological subtypes with different clinical behaviors. Although the stage at presentation is highly predictive of survival, none of the existing staging systems are ideal. The fact that local, regional, or distant recurrences can be seen despite maximum treatment further increases the importance of accurate staging and close imaging follow-up [2]. The rarity and histological and clinical diversity of these tumors present a diagnostic challenge.

Imaging has an essential role in evaluating the response to treatment and detecting recurrences, in addition to initial primary staging. Ultrasound (US), computed tomography (CT), and magnetic resonance imaging (MRI) are routinely used modalities for morphological evaluation and differentiation of neoplastic from inflammatory processes and benign and malignant tumors in the sinonasal region. Whole-body positron emission tomography (PET) combined with either CT or MRI provides functional information throughout the entire body in one session [3].

Depending on the tumor type, PET provides accurate staging of the primary tumor, nodal involvement, and distant metastasis of head and neck malignancies, including sinonasal cancers [4,5,6]. PET/CT contributes not only to the determination of initial staging, but also to the planning of treatment and surveillance. Chemoradiotherapy is commonly applied as the main treatment strategy for sinonasal malignancies, and PET/CT has a crucial role in monitoring the treatment response and detecting recurrences that may be missed by conventional anatomical imaging modalities and endoscopy [5,7].

The well-established workhorse of nuclear oncology is 2-[fluorine 18]fluoro-2-deoxy-d-glucose (^18^F-FDG), which assesses glucose metabolism [5,8,9]. Other important radiopharmaceuticals used in nuclear oncology for imaging sinonasal malignancies include somatostatin analogs, such as ^68^Ga-dodecanetetraacetic acid-Tyr3-octreotate (DOTATATE)/dodecanetetraacetic acid-D-Phe1-Tyr3-octreotate (DOTATOC), as well as ^11^C-choline [10,11,12,13,14,15,16].

In this review, the role of ^18^F-FDG PET/CT in diagnosis, initial staging, treatment planning, and assessment of the treatment response in various sinonasal tumors will be discussed along with demonstrative case examples. Additionally, radiotracers other than FDG and their utility in sinonasal malignancies will be covered.

In this review, our main aim was to discuss the role of PET/CT in diagnosis, initial staging, treatment planning, and assessment of the treatment response in various sinonasal tumors with demonstrative case examples. Additionally, recently used radiotracers other than FDG and their utility in sinonasal malignancies were covered. As we best know, there is no such detailed review of sinonasal tumors and their PET applications in the medical literature that emphasizes their unique diagnostic features, their importance in initial staging, their power in treatment planning, and their high accuracy in terms of treatment response.

## 2. ^18^F-FDG PET/CT Applications in Sinonasal Neoplasms

### 2.1. Malignant Lesions

#### 2.1.1. Sinonasal Squamous Cell Carcinoma

Squamous cell carcinoma (SCC) of the sinonasal tract is a malignant epithelial neoplasm arising from the surface epithelium lining the nasal cavity and paranasal sinuses. It is the most common malignancy of the sinonasal region and constitutes 40–60% of cases [17,18,19,20].

Risk factors for sinonasal SCC mainly reflect those valid for all head and neck subsites. These include tobacco use, alcohol consumption, and infection with human papillomavirus (HPV) [20,21,22]. Malignant degeneration of an inverted papilloma is also a well-established etiology of sinonasal SCC, with a reported transformation rate ranging from 1 to 10% [23,24,25].

The maxillary sinus is the most common site for sinonasal SCCs (≈60%), followed by the nasal cavity and ethmoid air cells, and rarely the frontal or sphenoid sinuses [26,27]. Tumors originating from the nasal cavity and paranasal sinuses each account for approximately half of the cases [28].

Depending on the location and stage of the disease, multimodality therapy is the mainstay of treatment of sinonasal SCCs [29]. Complete surgical resection, either with endoscopic endonasal or combined cranioendoscopic approaches, followed by adjuvant postoperative radiotherapy or concurrent chemoradiotherapy, is the standard treatment for SCC of the sinonasal tract. In advanced-stage disease, neoadjuvant chemotherapy has been advocated to provide better tumor control and orbital preservation [30].

By virtue of SCC’s origin in midline structures, such as the nasal septum, contralateral or bilateral lymphadenopathy may be observed [31]. The incidence of lymph node metastasis at presentation is approximately 15%, while simultaneous distant metastasis is rare. Even with a successful combination therapy, the overall clinical outcome is poor, with a 5- and 10-year overall survival rate of 30% and 21%, respectively [26]. In addition to the poor prognosis, patients older than 50, particularly African American patients and/or those who present with an advanced disease stage, have particularly unfavorable outcomes [26].

CT is the imaging modality of choice in terms of demonstrating structural bone changes, such as thinning or destruction, owing to its high spatial resolution and capability of detecting bone density alterations [32,33,34]. A sinonasal soft tissue mass with bone destruction is a classical scenario of SCC that may be seen on CT. On the other hand, SCCs also have nonspecific MRI features consisting of an isointense signal in T1-weighted imaging, a hypo-to-intermediate signal in T2-weighted imaging (compared to fluid), and variable enhancement on contrast-enhanced images. The enhancement of the SCCs is often less than that of the adjacent sinus mucosa. Smaller lesions are typically homogeneous in signal intensity, while the larger ones are commonly more heterogeneous with areas of necrosis and hemorrhage. In particular, inverted papillomas and lymphomas may show similar MRI findings, such as skull base invasion and destruction. In addition, spreading into the anterior and middle cranial fossae with or without dural involvement is common [35]. Contrast-enhanced MRI is the imaging modality of choice for structural imaging and is crucial in identifying the soft tissue involvement, both at the sinonasal region boundaries [36,37,38] and in adjacent regions, such as the brain, orbital contents, cavernous sinus, and internal carotid artery [39,40,41]. It has also been shown that sinonasal SCCs have higher apparent diffusion coefficient (ADC) values compared to sinonasal lymphoma [42]. In addition, perineural involvement is also a common feature of sinonasal SCC, and evaluation of the entire course of the cranial nerves is strongly advised to detect skip lesions. As mentioned above, sinonasal SCCs may sometimes originate from an inverted papilloma, constituting an additional dilemma for differential diagnosis. From this point of view, MRI plays an invaluable role in differentiating benign inverted papillomas from SCC [43,44]. Convoluted cerebriform patterns and lower ADC values [44,45], especially when associated with an increased maximum standard uptake value (SUVmax) on ^18^F-FDG-PET/CT, are associated with a higher probability of malignancy [43,46]. Yilmaz et al. [46] found a statistically significant difference in terms of SUVmax levels between nasal polyps, inverted papillomas, and SCC in their study, which included 27 patients with mean SUVmax values of 2.9, 7.8, and 17.8, respectively. However, these results could not be validated in some of the other studies [47,48].

**Figure 1:**
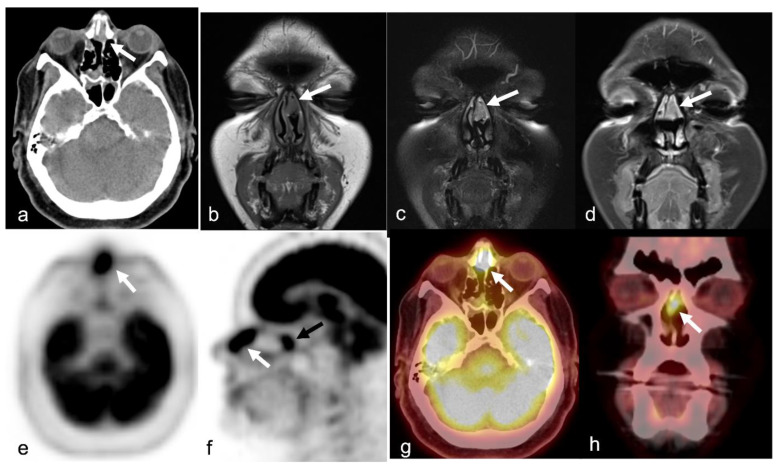
Sinonasal squamous cell carcinoma. A 49-year-old female with a history of nasal obstruction and occasional nose bleeds for a few months. The axial unenhanced CT image (**a**) demonstrates a left anterior nasal mass (white arrow). On corresponding coronal MRI images (**b**–**d**), the mass that is isointense relative to muscles on T1w (**b**) is heterogeneously hyperintense relative to muscles on fat-saturated T2w images (**c**), showing homogeneous enhancement on postcontrast T1w fat-saturated (**d**) images (white arrow). Attenuation-corrected axial (**e**), sagittal (**f**) PET, and axial (**g**) and coronal (**h**) fused PET/CT images demonstrate high FDG metabolism of the lesion (white arrow). On image (**f**), a surgically proven second squamous cell carcinoma focus on the left posterior nasal cavity is seen (black arrow).

Since it allows simultaneous acquisition of anatomical and metabolic data from primary tumors, provides an accurate diagnosis of regional neck metastases, and enables the identification of distant metastases in a single session, ^18^F-FDG PET/CT is a principal tool for the initial staging of advanced head and neck SCCs, including sinonasal SCCs [49]. SCCs mostly demonstrate moderate-to-intense uptake on ^18^F-FDG PET/CT imaging [50] (Figure 1). Ozturk et al. [51] reported the SUVmax, SUVmean, and SUVratio-mediastinum of the sinonasal SCCs as 17.9 ± 8.1 (±SD), 8.6 ± 3 (±SD), and 7.6 ± 3.2 (±SD), respectively. They concluded that, despite overlaps in some of the rare subtypes, SUV levels may be used as a “metabolic biopsy” for differentiating the various histopathologic subtypes of the sinonasal malignancies because they may help in the characterization of some of the more common ones [51]. As with other head and neck SCCs, the interval decrease or resolution of uptake at the first post-treatment scan is a strong predictor of better overall survival [51]. The intensity of uptake of ^18^F-FDG allows appraisal of tumor aggressiveness, which is a predictor of prognosis. Higher SUV values in head and neck SCC are associated with a worse prognosis [52].

It is important to distinguish SCC from other common histological types of sinonasal malignancies. Kim et al. [53] reported on features of sinonasal non-Hodgkin lymphoma (SN-NHL) and SCC, showing that, in contrast to SCC, SN-NHL lesions tend to present a bulky lesion with bone destruction, a homogeneous signal on T2-weighted and postcontrast images, and low ADC values without adjacent bone destruction. Both tumor types showed a high degree of enhancement. However, the study showed no statistically significant difference between cervical lymphadenopathy, Waldeyer’s ring involvement, or ^18^F-FDG-PET/CT SUVmax between these two tumors. Regarding other sinonasal malignancies, differentiation can be difficult, such as with sinonasal adenocarcinoma, which is essentially indistinguishable from SCC by imaging features on CT, MRI, or ^18^F-FDG-PET/CT [41].

#### 2.1.2. Sinonasal Lymphoma

Sinonasal NHL (SN-NHL) is the second most common sinonasal malignancy after SCC [54]. The two most frequent subtypes are diffuse large B-cell lymphoma (the most common) and natural killer (NK)/T-cell lymphoma [55]. Diffuse large B-cell lymphoma (DLBCL) has a predilection for the sinuses, particularly the maxillary sinuses, followed by the nasal cavity [56]. The treatment of sinonasal lymphoma is nonsurgical, with chemotherapy being the mainstay of treatment for most patients and radiation mainly used for invasive or refractory lesions and, increasingly, in NK/T-cell lymphoma [55].

Staging is used to determine pretreatment risk stratification and the selection of therapy. CT is often the initial examination method for sinonasal masses, but MRI is more sensitive than CT for the detection of lymphoma, which usually presents as a tumor or mass with soft tissue attenuation [57]. Using conventional MRI, sinonasal NK/T cell lymphoma is more often located in the nasal cavity, has ill-defined margins, and shows heterogeneous signal intensity, internal necrosis, and marked enhancement [58]. On the other hand, DLBCL was more often located in the paranasal sinuses with intracranial or orbital involvement, with homogenous intensity and mild enhancement [58]. The tumor usually demonstrates intermediate signal intensity on T1-weighted images and variable signal intensity on T2-weighted images without fat saturation [59]. T2-weighted images have been shown to be useful in differentiating tumors from normal mucosa, a significant advantage over CT. The tumors typically show a restricted diffusion pattern with high signal intensity on diffusion-weighted imaging (DWI) and low signal on ADC [60,61]. The ADC value can be used to differentiate between lymphoma and SCC with an accuracy of 98% [62].

**Figure 2:**
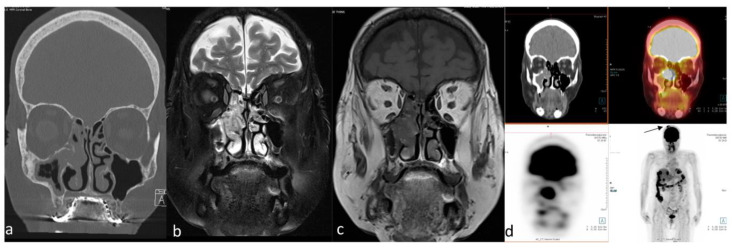
Sinonasal involvement by lymphoma. An 84-year-old female patient with a past medical history of rheumatoid arthritis presenting with an ulcerative scalp lesion. The biopsy of the scalp lesion was consistent with EBV (+) DLBCL. Paranasal sinus CT (**a**) shows a right nasal cavity lesion expanding the right ostiomeatal unit and extending into the right maxillary sinus. Post-obstructive frothy secretions in the right maxillary sinus are noted. Fat-saturated coronal T2w (**b**) and non-fat-saturated coronal T1w (**c**) images better depict the primary lesion as mildly T2w hyperintense areas, whereas mucosal disease is more T2w bright. ^18^F-FDG PET/CT images (**d**) demonstrate the intense avidity of the right nasal cavity lesion as well as the scalp lesion (arrow) and two FDG-avid right pulmonary lesions.

Since the 1990s, ^18^F-FDG has played an important role in the management of lymphoma. Most SN-NHLs have a high glucose metabolism, so ^18^F-FDG PET/CT is an accurate method for diagnosis, staging, and therapeutic monitoring [63,64,65,66,67] (Figure 2). Thus, a PET-CT scan indicating bone or marrow involvement is usually sufficient to designate advanced-stage disease, and a bone marrow biopsy is not required [68].

Regarding NK/T-cell lymphoma specifically, ^18^F-FDG-PET/CT data are sparse. In a study conducted on 15 patients diagnosed with NK/T-cell lymphoma, the uptake of ^18^F-FDG was found to be similar between patients in stages 1 and 3. Two of the 15 patients were found to have an indeterminate diagnosis, for which ^18^F-FDG-PET/CT was used to guide a biopsy. In the remaining 13 patients with a definite diagnosis, the use of ^18^F-FDG-PET/CT changed the staging in 6 patients, downstaging 2 patients and upstaging 4 patients [69]. In another larger study on 52 patients with NK/T-cell lymphoma, the detection rate for nodal and extranodal lesions was compared between ^18^F-FDG PET/CT and CT and MRI (PET: 58% and 69%; CT and MRI: 44% and 61%). Furthermore, ^18^F-FDG PET/CT scans were generally more accurate than the other conventional imaging modalities in staging those patients [70].

#### 2.1.3. Sinonasal Adenocarcinoma

Adenocarcinoma accounts for 10–20% of all sinonasal cancers [1,71,72] and represents the most common mucosal epithelial malignancy in Europe [1]. The ethmoid sinuses are the most common region for sinonasal adenocarcinomas, followed by the nasal cavity, maxillary antrum, and olfactory region [73,74,75]. There are certain predisposing risk factors, especially for intestinal-type adenocarcinomas, including chronic occupational exposure to hardwood dust, formaldehyde, chrome pigment, clothing dyes, and leather dust, which explains the male predilection of the disease [21,76]. This fact is also a reason for the multifocality of the tumor detected in different mucosal areas of the nasal cavities, even those that are distant from each other.

About two-thirds of naso-ethmoidal adenocarcinoma recurrences develop in field, and nodal and distant metastases account for 20% and 10%, respectively. The risk of local recurrence for adenocarcinomas is statistically related to pathologic T (pT) stage and dural invasion. Leptomeningeal metastases in intestinal-type adenocarcinomas have been reported at diagnosis or during follow-up [77,78,79].

Standard management for intestinal-type adenocarcinoma is surgery followed by radiation therapy. Single-modality treatment with surgery alone is recommended for early-stage (pT1) and low-grade adenocarcinomas [80]. Considering the possibility of tumor spreading to the leptomeninges at the time of diagnosis or late in follow-up, prophylactic brain irradiation may be considered in high-grade lesions with intracranial invasion [81]. Since the risk of regional metastasis is low (7%) in sinonasal adenocarcinoma, elective treatment of neck lymph nodes is not routinely undertaken [1].

Although the general imaging features of adenocarcinomas may overlap with those of other sinonasal tumors, especially squamous cell carcinoma, the location of the tumor is one of the most helpful distinguishing features. Adenocarcinomas prefer the ethmoid sinuses, while squamous cell carcinomas arise in the maxillary antrum and nasal cavity. CT and MRI can provide information regarding tumor size, extent, and depth of invasion into adjacent anatomical structures, which is crucial for surgical and therapeutic planning. CT is the modality of choice for evaluating the adjacent bone structures. The pattern of bone invasion may help predict tumor aggressiveness. Benign lesions and low-grade malignancies may show smooth osseous expansion secondary to their slow and expansile growth patterns. On the other hand, high-grade malignancies mostly cause bone destruction [72]. Sinonasal adenocarcinomas are mostly seen as a soft tissue mass, occasionally with intralesional calcifications on CT images. Bulging of the nasal septum towards the midline and widening of the olfactory cleft can be seen in unilateral olfactory cleft adenocarcinomas [82]. Potential extension to the skull base and frontal lobes is generally observed in ethmoid sinus adenocarcinomas [83].

MRI with gadolinium is the modality of choice for the assessment of sinonasal tumors due to its superior soft tissue resolution. Sinonasal adenocarcinomas usually demonstrate an isointense signal on T1-weighted MR images, while T2-weighted signal intensity varies depending on cellularity and mucin content. On T2-weighted imaging, paucicellular tumors can be hyperintense, whereas cellular tumors may show moderate signal intensity. The higher mucin production is related to the high signal intensity on T2-weighted imaging, whereas adenocarcinomas with no or limited mucin production tend to be iso-to hypointense on T2-weighted imaging [72]. Hyperintense areas within the tumor on T1-weighted images most likely represent hemorrhage, melanin, lipid, protein, or mineral elements [72]. Adenocarcinomas tend to develop more avid enhancement and have better defined borders than squamous cell carcinomas on postcontrast images [84,85]. An irregular signal void of the osseous cortex is often an indicator of invasion by the tumor. Fat-suppression techniques have been developed to define intracranial extension and differentiate between cystic and solid lesions. MRI is crucial to the identification of perineural spread. DWI with ADC measurement is a helpful technique to distinguish benign and malignant sinonasal tumors [72,86,87,88].

^18^F-FDG PET/CT provides information about tumor cell viability and tumor behavior. Whole-body ^18^F-FDG PET/CT is superior to conventional imaging with MRI or CT in evaluating primary tumors, regional lymph nodes, distant metastases, and potential second primary tumors all within a single examination [89]. ^18^F-FDG PET/CT can sometimes detect hypermetabolic metastatic foci that cannot be seen on conventional imaging [90]. The sensitivity of ^18^F-FDG PET/CT to detect disease at the primary site ranges from 94–100% [8,9]. ^18^F-FDG PET/CT is also an important tool for tumor staging and surveillance following definitive treatment, particularly to distinguish local recurrence from postoperative changes [5,8,9,85,91,92].

**Figure 3:**
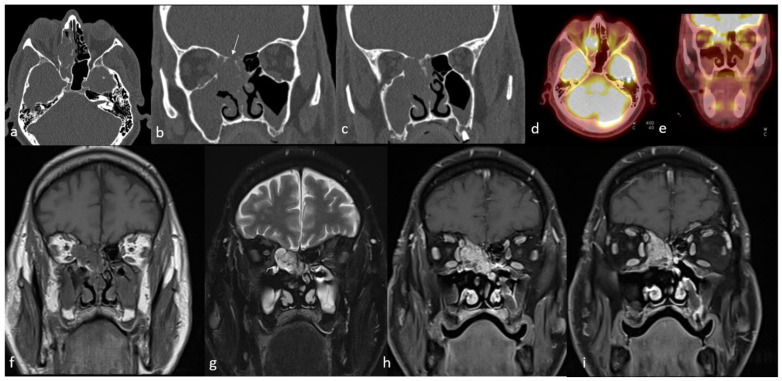
Sinonasal adenocarcinoma. A 62-year-old man with a history of chronic sinus disease presenting with right-sided epistaxis. Initial CT (**a**–**c**) shows an expansile right nasal cavity mass centered in the superior/supreme meatus as well as involving the right middle turbinate. Right extraconal extension through lamina papyracea dehiscence is present. There is also dehiscence along the cribriform plate (arrow, (**b**)). Axial (**d**) and coronal (**e**) fused ^18^F-FDG PET/CT images demonstrate a hypermetabolic right ethmoid sinus mass with no evidence of lymphadenopathy or distant metastatic disease. Coronal T1w (**f**), T2w (**g**), and post-contrast T1w (**h**) show a heterogeneously enhanced right ethmoid sinus lesion with extraconal extension. The more anterior post-contrast T1w (**i**) image does not demonstrate abnormal leptomeningeal enhancement despite the underlying bone dehiscence. Subsequent pathology showed an intestinal type of adenocarcinoma with a mucinous growth pattern.

Sinonasal adenocarcinomas may demonstrate moderately high metabolic activity, similar to other common sinonasal malignant tumors [51] (Figure 3). Ozturk et al. [51] analyzed a variety of sinonasal primary tumors in 97 patients using several semiquantitative measures of uptake on ^18^F-FDG PET/CT [51]. The measures were SUVmax, mean standardized uptake value (SUVmean), and the ratio of the SUVmax of the primary tumor to the SUVmean of the mediastinal blood pool (SUVratio-mediastinum). For primary sinonasal adenocarcinoma, SUVmax, SUVmean, and SUVratio-mediastinum were 9.8 ± 6 (±SD), 4.8 ± 2.3 (±SD), and 3.3 ± 1.6 (±SD), respectively [51]. These values were significantly lower than for squamous cell carcinoma, which showed SUVmax, SUVmean, and SUVratio-mediastinum values of 17.9 ± 8.1 (±SD), 8.6 ± 3 (±SD), and 7.6 ± 3.2 (±SD), respectively [51]. Felix-Ravelo et al. [93] evaluated ^18^F-FDG uptake in six types of sinonasal malignancies, most commonly adenocarcinoma, in the studied population. This study found that sinonasal undifferentiated carcinoma (SNUC) had the highest mean SUVmax of 14.2, followed by adenocarcinoma with a mean SUVmax of 9.9 [93].

Whole-body ^18^FDG PET/CT is a very useful method as a screening tool for the detection of distant metastasis and regional lymphatic metastases in sinonasal neoplasms and may be performed as part of the routine pretreatment evaluation of metastatic workup [8,9,94]. Though data are limited, the general approach to imaging and staging is similar to that for sinonasal squamous cell carcinoma [95]. For sinonasal tumors in general, ^18^FDG-PET/CT has high sensitivity and specificity for identifying metastatic lymph nodes and distant metastases, with sensitivity and specificity estimated at 83% and 96%, respectively, for nodes and 81% and 99%, respectively, for distant metastatic disease [94]. ^18^FDG-PET/CT is also a useful tool to detect tumor recurrence [8,96].

Postsurgical PET/CT findings are highly prognostic in patients with sinonasal malignancies. Negative findings on the first posttreatment ^18^FDG-PET/CT scan predict significantly better overall survival [97]. Abu-Ghanem et al. [97] reported that the overall 5-year survival was statistically significantly lower in the first postsurgical ^18^FDG-PET/CT-positive group (35%), compared to the negative group (93%).

Although most sinonasal malignancies appear to be ^18^F-FDG avid, in cases of low SUVmax, other radiotracers could be considered, such as ^18^F-*Dihydroxyphenylalanine* (DOPA) and ^18^F-Fibroblast activation protein (FAPI), although they are not widely available or are investigational at this time [98,99].

#### 2.1.4. Sinonasal Mucosal Malignant Melanoma

Primary sinonasal mucosal malignant melanoma is an uncommon entity, accounting for 0.3% to 2% of all malignant melanomas and 4% to 10% of melanomas in the head and neck region. Melanomas constitute 4% to 7% of all sinonasal tumors [100,101,102].

The head and neck region is the most common place involving mucosal malignant melanoma, followed by the rectum and the anus, the urinary system, and the female genitalia [103,104]. In the head and neck, the nasal cavity, especially the nasal septum, lateral nasal wall, middle and inferior turbinates, oral cavity, and, less frequently, paranasal sinuses, especially the maxillary antrum and the ethmoid sinuses, are the most prevalent sites [101,103,105,106].

The most important factor in determining the prognosis and outcome of sinonasal mucosal malignant melanoma is the metastatic status [101]. Dreno et al. [107] estimated the 3-, 5-, and 15-year overall survival rates of primary sinonasal mucosal melanomas at 50%, 33%, and 14%, respectively. Recurrent disease peaks approximately 1 year after the initial diagnosis [100,108,109,110,111,112,113,114]. In approximately one-third of head and neck mucosal malignant melanomas, lymph node metastases are usually noted at first presentation, which signifies a worse prognosis [103,115]. On the other hand, recurrence in the regional lymph nodes is rare [116,117,118].

Aggressive surgery for local control of sinonasal mucosal malignant melanoma remains the mainstay of treatment but may be challenging due to the involvement of adjacent vital structures in the head and neck, anatomical site-related limitations, and lentiginous, multifocal growth patterns [119,120,121]. Local recurrence is also commonly observed, even when the margins are free of tumor [122,123]. Adjuvant radiotherapy has been shown to improve loco-regional control in various studies, but it has limited or no significant benefit in terms of survival [124,125,126]. Although several trials have demonstrated durable response rates of 30–40%, the exact response rates are unknown for sinonasal mucosal malignant melanoma to immune checkpoint inhibitors, such as pembrolizumab or nivolumab, which are classically used to treat cutaneous melanoma [127,128,129]. Nemvaleukin alfa, which is an engineered interleukin-2 variant, is also another potential therapeutic agent that has been actively studied in recent years. Fast Track designation was recently granted by the Food and Drug Administration (FDA) for the treatment of patients with mucosal melanoma who had previously been treated with checkpoint inhibitors, based on several responses observed in an earlier phase I study [130].

CT and MRI are the main imaging modalities for understanding the anatomy and local tumor involvement [131]. On CT, despite the aggressive biological behavior of the tumor, it may appear as a well-defined polypoid mass exerting a pressure effect with well-defined bone remodeling. While a CT examination is usually required to assess potential bone erosion, MRI often provides better tumor and soft tissue differentiation [132]. Sinonasal mucosal malignant melanoma is commonly seen as a solid soft tissue mass with local invasion on MRI at presentation. MRI seems to be the imaging modality of choice for assessing possible dural or intracranial involvement. MRI must be acquired in addition to CT whenever orbital or skull base infiltration is suspected [133]. The appearance of the tumor depends on the amount of melanin in it [100,131,134]. Intratumoral melanin and hemorrhage may show T1-weighted hyperintensity due to paramagnetic properties. Contrast enhancement is variable, and complete characterization is difficult due to the natural T1 shortening effect of melanin in many of these tumors. Amelanotic sinonasal melanomas may show low signal intensity on T1- and T2-weighted images [134].

^18^F-FDG PET/CT is increasingly used for initial staging for head and neck malignancies [135] and is superior to CT alone for staging and evaluation of therapy response in many oncological diseases, including mucosal malignant melanoma [136,137,138]. ^18^F-FDG PET/CT provides beneficial information in terms of surgical therapy planning, especially if the tumor is in a challenging location, such as in the nasal cavity or paranasal sinuses and close to vital structures [133].

**Figure 4:**
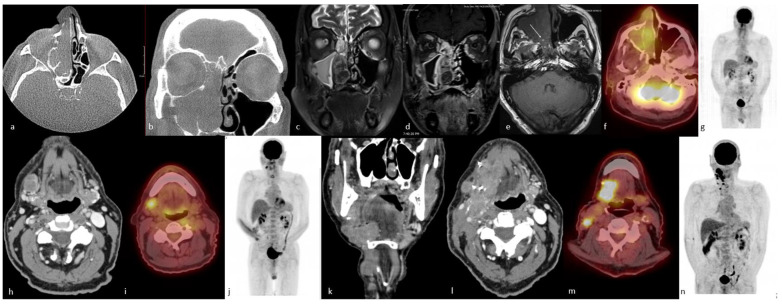
Nasal cavity malignant melanoma. Initial non-contrast CT images (**a**,**b**) show an expansile, destructive soft tissue lesion in the right nasal cavity, resulting in destruction of the right turbinates and ethmoid septae. The right medial maxillary sinus is dehiscent, with opacification of the right maxillary sinus. The cribriform plate is intact. Coronal fat-saturated T2w and post-contrast T1w images (**c**,**d**) show an enhancing mass primarily in the right nasal cavity without nodular dural enhancement to suggest intracranial extension. The axial non-contrast T1w image (**e**) shows focal areas of hyperintensity (arrow), which may represent mucinous components, melanin, or hemorrhagic content. Subsequent ^18^F-FDG PET/CT ((**f**)—axial fused, (**g**)—MIP) shows FDG avidity of the primary lesion without distant metastasis. A biopsy revealed malignant melanoma. The patient underwent endoscopic resection, medial maxillectomy, and total ethmoidectomy and completed radiotherapy. Follow-up CT (**h**) and ^18^F-FDG PET/CT ((**i**)—axial fused, (**j**)—MIP) show new necrotic right submandibular lymphadenopathy without sign of local recurrence at the right nasal cavity. The patient underwent a subsequent selective right neck dissection. Follow-up CT (**k**,**l**) and ^18^F-FDG PET/CT ((**m**)—axial fused, (**n**)—MIP) 6 months after neck dissection revealed lymphadenopathy in the right neck, including conglomerate lymphadenopathy in the submental, submandibular, and sublingual spaces and the root of the tongue.

Although ^18^F-FDG PET/CT is well established in the assessment of cutaneous melanoma, its role in sinonasal mucosal malignant melanoma is recently emerging [133,135,139,140,141,142,143]. Haerle et al. [64] reported 10 patients with sinonasal mucosal malignant melanoma who underwent CT, MRI, and ^18^F-FDG PET/CT imaging at staging and follow-up, with ^18^F-FDG PET/CT successfully detecting all primary tumors and regional and distant metastases except for one cerebral metastasis [64]. ^18^F-FDG PET/CT is important in the surveillance of mucosal malignant melanoma and the detection of distant recurrence (Figure 4). It should be performed at least once in the postoperative period or more frequently for distant metastasis screening, given the tendency for distant metastasis [133]. Differentiating scar tissue from viable tumors, especially in recurrent tumors, is not possible with CT alone [133]. Roth et al. [105] showed that while local tumor control in sinonasal mucosal malignant melanomas can be achieved in many cases, the majority of the patients died from distant metastasis, which supports the use of ^18^F-FDG PET/CT during the follow-up period.

Quantitation of tumor uptake in ^18^F-FDG PET/CT imaging can be expressed in various ways, with SUVmax, SUVmean, and various SUV ratios depending on the organ of reference (mediastinal blood pool or liver). Haerle et al. showed significant ^18^F-FDG uptake (SUVmax > 4) on baseline imaging in 6/10 patients), similar to findings by Samstein et al. [118]. Ozturk et al. [51] reported FDG uptake in untreated sinonasal mucosal malignant melanoma with SUVmax, SUVmean, and SUVratio-mediastinum 20.8 ± 18.2 (±SD), 8.4 ± 5.1 (±SD), and 6.4 ± 4.9 (±SD), respectively. Mucosal malignant melanomas had the second highest SUVmax after SNUC. In a similar study with a smaller study group, Felix-Ravelo et al. [93] found the SUVmax and SUVratio-liver of the sinonasal mucosal malignant melanoma lesions to be 7.0 ± 3.8 (±SD), and 2.5 ± 1.0 (±SD), respectively. There was an overlap in degrees of uptake by various tumor types, with similar uptake noted by sinonasal mucosal malignant melanoma, adenoid cystic carcinoma, and olfactory neuroblastoma. Inubishi et al. [139], in their study of 13 patients, found a significant relationship between FDG avidity before carbon ion radiotherapy, and overall survival and distant metastasis.

The limitations of ^18^F-FDG PET/CT imaging are as follows: (1) poor detection of brain metastases due to the high background metabolism; (2) poor detection of small melanoma metastases due to size below the detection threshold for PET; (3) inability to differentiate melanotic from amelanotic variants; and (4) poor detection of subtle liver lesions due to physiologic background tracer [136,144,145]. With modern ^18^F-FDG-PET/CT scanners, the theoretical three-dimensional spatial resolution is about 4 mm; however, 6–8 mm is more realistic due to various factors, such as motion and the tumor-related uptake ratio [146].

^18^F-FDG PET/CT may be less useful in differentiating complete response from partial response with residual tumor burden or differentiating residual tumor from inflammation. An investigational PET radiotracer, 30-deoxy-30-[18F]fluorothymidine (FLT), a nucleoside analog, has been developed as a marker of cellular proliferation [146]. Shields et al. [146] showed that ^18^F-FLT PET/CT imaging was beneficial for predicting the therapeutic outcome of patients with head and neck mucosal malignant melanoma, and also identified some prognostic factors among the clinical parameters, although the subjects represented a particular subpopulation treated with carbon ion radiotherapy.

^18^F-FDG PET/MRI is the most sophisticated cross-sectional imaging modality for initial staging and restaging of sinonasal mucosal malignant melanomas because of its ability to obtain detailed anatomical and metabolic information regarding loco-regional tumor extent and distant disease, including brain metastases, in one single examination [147]. Meerwein et al. [147] reported that ^18^F-FDG PET/MRI provided new possibilities in the radiological evaluation of sinonasal mucosal malignant melanomas and immunotherapy, resulting in substantial progression-free survival in selected cases. Kuhn et al. [148] showed in their study comparing ^18^FDG PET/CT with ^18^FDG PET/MRI in head and neck cancers that ^18^FDG PET/MRI provided superior accuracy in distinguishing the tumor tissue from entrapped mucus in sinonasal cavities and evaluating the perineural spread. The same study showed similar accuracy between ^18^FDG PET/MRI and CT in detecting bony metastases, such as in the skull base. Another study showed that ^18^FDG PET/MRI may become a “one-stop-shop whole-body N- and M-staging tool” in high-risk melanoma patients. ^18^FDG PET/MRI has some particular advantages compared to the traditional approach, including separate ^18^FDG PET/CT and MRI studies, in addition to the lower radiation exposure owing to the absence of the CT component [149]. A study by Sekine et al. [150] addressing the initial staging of head and neck tumors provided a high diagnostic accuracy of ^18^FDG PET/MRI that was at least equal to ^18^FDG-PET/CT, with the added advantage of better soft-tissue contrast, which is of particular importance for the assessment of dural or orbital involvement. In addition to being a single examination, ^18^FDG PET/MRI has advantages over two separate scans in terms of total scan time, patient flow, and potential cost [151].

#### 2.1.5. Sinonasal Undifferentiated Carcinoma

Sinonasal undifferentiated carcinoma (SNUC) is an uncommon, high-grade, and aggressive tumor that accounts for approximately 3–5% of all sinonasal cancers [20,72,152,153]. SNUC is commonly considered a diagnosis of exclusion [152]. The distinction between SNUC and olfactory neuroblastoma (ONB) is crucial due to significant differences in clinical behavior, prognosis, and treatment strategies. While SNUC shows rapid growth with a poor prognosis, ONB mostly shows slow growth with a better prognosis [154].

This tumor most commonly originates from the superior nasal cavity and the ethmoid sinus [72,153]. Orbital and intracranial invasion, nodal involvement, and distant metastasis are common findings [72]. Most patients are diagnosed in advanced stages at presentation, with a propensity for early metastatic disease [155]. At first presentation, 67–81% of patients have locally advanced (T4) disease, and 13–21% of them show lymph node metastases [156,157,158]. The 5-year survival rates are variable, ranging between 6 and 74% [29,159,160,161,162,163,164,165]. The recurrence rate ranges between 42 and 50% [164,165,166]. The time to recurrence ranges from 3–33 months [165].

Treatment of SNUC remains challenging, with poor survival outcomes [165]. Definitive surgical resection can be performed in cases without diffuse intracranial involvement and without distant disease [167]. On the other hand, surgery followed by chemoradiotherapy or radiotherapy alone may be applied to patients with early-stage disease [168].

There are several significant imaging features that can help differentiate SNUC from other sinonasal malignancies. These tumors demonstrate more frequent distant or nodal metastases with more aggressive and invasive features on CT [85,153]. CT typically shows a noncalcified mass with variable contrast enhancement and a necrotic center [72,85]. SNUCs frequently result in osteolysis in the adjacent bone, such as SCC, aggressive sarcomas, and metastatic disease [84]. On MRI, they display a nonspecific imaging appearance [84], are observed as isointense on T1w and iso- to hyperintense on T2w, and demonstrate heterogeneous enhancement on contrast-enhanced T1w imaging [84,85]. As in other sinonasal tumors, MRI is very helpful in identifying perineural tumor spread (PNS) and extension to the skull base, orbit, and intracranial areas [84]. It has been shown that SNUC has a lower ADC value than adenoid cystic carcinoma but a higher FDG than ONB [45,169].

**Figure 5:**
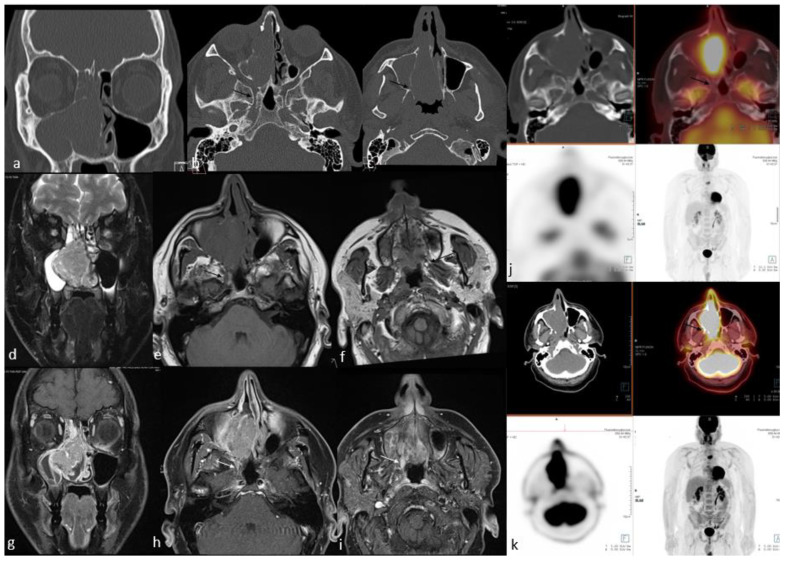
Sinonasal undifferentiated carcinoma. A 42-year-old male patient presenting with progressive symptoms of postnasal drainage, nasal congestion, and facial pressure. Initial CT (**a**) shows an expansile right nasal cavity lesion with medial bowing of the nasal septum and lateral bowing of the maxillary sinus wall. There is soft tissue density opacification in the right maxillary sinus; however, it is not possible to discern whether this represents postobstructive opacification or lesion extension. Coronal fat-saturated T2w (**d**) and post-contrast T1w (**g**) images better delineate the lesion and postobstructive mucosal opacification. Axial CT image (**b**) at the level of the vidian canal shows asymmetric enlargement of the right vidian canal (arrow, (**b**)) with asymmetric soft tissue intensity on the non-fat-saturated axial T1w (**e**) image and asymmetric enhancement on the post-contrast image (**h**,**i**). The ^18^F-FDG PET/CT image (**j**) at the same level shows FDG avidity of the primary nasal cavity lesion without uptake at the right vidian canal. More caudal axial CT (**c**) shows erosive changes in the right pterygoid plates with erosion of the greater palatine canal (arrow, (**c**)). There is loss of normal fat planes in the right greater palatine canal on the axial T1w image (**f**), with preservation of fat in the contralateral palatine canal (arrow, (**g**)). The postcontrast axial image shows asymmetric enhancement in the right greater palatine canal (arrow, (**h**)). ^18^F-FDG PET/CT image (**k**) at the same level shows FDG uptake extending into the right greater palatine canal. Pathology was consistent with high-grade carcinoma, most consistent with sinonasal undifferentiated carcinoma.

PET/CT is a functional imaging modality that is highly sensitive for detecting primary and recurrent malignant tumors in the head and neck [170]. This technique is usually performed during the initial staging work-up of head and neck malignant tumors due to the high sensitivity of small malignant foci and its high effectiveness in detecting cervical nodal metastasis [171] (Figure 5). Wu et al. [172] reported that the SUVmax obtained from ^18^F-FDG PET/CT studies may help with distinguishing the ONB and SNUC. This pilot study concluded that the SUVmax of SNUCs is approximately five times higher than the SUVmax of ONBs. Elkhatib et al. [169] also reported similar results regarding SUVmax of ONB and SNUC, and they advocated that these two tumors could be differentiated by looking at SUVmax levels (mean SUVmax: 35.6 and 7.2 in SNUC and ONB, respectively). The same study also advocated that this SUVmax difference on ^18^F-FDG PET/CT may reflect the increased metabolic activity and aggressiveness of SNUCs [169]. In a similar study regarding the SUVmax of the common sinonasal malignant tumors, Felix-Ravelo et al. [93] found that the SUVmax and SUVratio (ratio of the SUVmax of the primary tumor and the SUVliver) of the SNUCs were 14.2 ± 5.8 (±SD) and 5.0 ± 3.0 (±SD), respectively. These were significantly higher than all other sinonasal malignancies, including SCC, adenocarcinoma, melanoma, neuroendocrine tumor, adenoid cystic carcinoma, mucosal malignant melanoma, and ONB.

#### 2.1.6. Sinonasal Adenoid Cystic Carcinoma

Adenoid cystic carcinoma (ACC) is a rare malignancy that most commonly arises from the salivary glands. It accounts for only 1% of head and neck cancers and 10% of all salivary gland tumors [173]. Sinonasal ACC has worse outcomes than other head and neck malignancies due to its advanced stage at diagnosis, involvement of the base of the skull, and likelihood of an incomplete resection [174]. Unlike other malignancies of the head and neck, smoking and alcohol are not associated [173]. Radical surgical resection for localized disease is the primary treatment modality. Patients who are female, N1-stage, or older than 79 years without distant metastases postoperatively could benefit from adjuvant radiotherapy [74].

**Figure 6:**
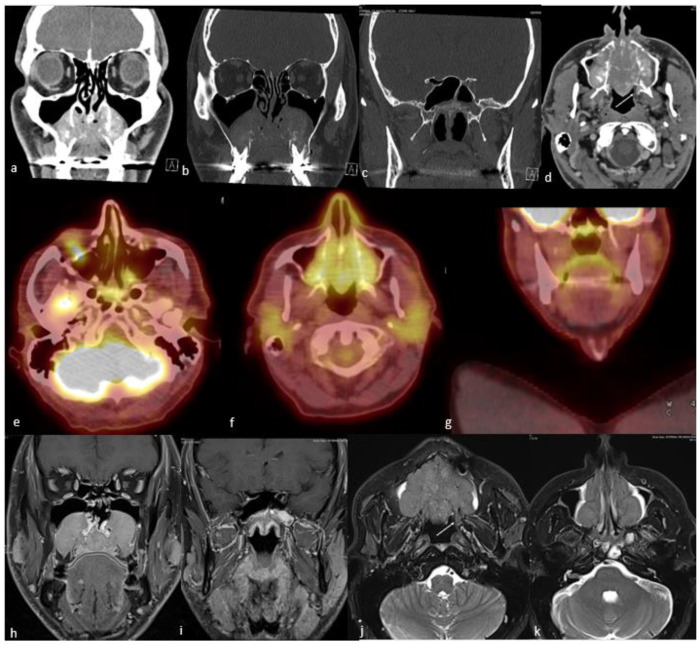
Sinonasal adenoid cystic carcinoma. A 46-year-old male presenting with chronic nasal congestion that had been progressive lately. The patient underwent septoplasty without prior imaging. During surgery, a large bulk mass filling the septal bone was found. Biopsy results were consistent with adenoid cystic carcinoma. CT after septoplasty (**a**–**d**) shows an expansile, destructive soft tissue lesion in the nasal cavity, scalloping the hard palate and extending into the bilateral maxillary sinus through erosive changes in the medial maxillary sinus walls. The more posterior coronal image (**c**) at the sphenoid sinus level shows soft tissue thickening in the floor of the sphenoid sinus with rarefaction of the inferior wall. The axillary slice at the level of the pterygoid plate (**d**) shows suspicious soft tissue thickening in the left greater palatine canal (arrow). Axial and coronal fused ^18^F-FDG PET/CT images (**e**–**g**) show hypermetabolism in the sphenoid floor soft tissue thickening (**e**,**g**) and the nasal cavity lesion extending into the maxillary sinuses (**f**). Post-contrast T1w images (**h**,**i**) demonstrate homogeneous enhancement of the nasal cavity lesion extending into the maxillary sinuses (**h**) and sphenoid floor soft tissue thickening (**i**). The axial T2w image at the level of the pterygoid plate (**j**) confirms the CT findings and demonstrates perineural spread along the left greater palatine nerve (arrow). The more cranial T2w image at the level of the pterygopalatine fossa (**k**) demonstrates normal fat planes without soft tissue thickening, indicating limited perineural tumor spread radiologically.

Conventional imaging with CT and MRI is the most frequently used modality for morphological characterization of ACC, but ^18^F-FDG PET/CT is also useful for staging and surveillance [175] (Figure 6). In a retrospective study conducted on 23 patients with ACC, ^18^F-FDG PET/CT added significant information to MRI and CT, detecting ACC primary sites with 87% sensitivity and upstaging 19% of patients compared to conventional imaging [176]. In another study of 36 patients with ACC, the addition or combination of ^18^F-FDG-PET/CT to MRI improved the detection of local tumors and metastatic spread and was found to be beneficial in restaging [177]. ^18^F-FDG PET/CT has higher sensitivity and diagnostic accuracy as compared to MRI for staging loco-regional tumor extension [177]. In one study, the sensitivity, specificity, and accuracy of detection of local tumors on ^18^F-FDG PET/CT versus MRI were reported at 96% versus 89%, 89% versus 89%, and 94% versus 89%, respectively [176].

Prostate-specific membrane antigen (PSMA) is a highly expressed transmembrane protein in prostate cancer cells and is often used for imaging and treatment of patients with prostate cancer [178]. However, it is also expressed in the neovasculature of many other solid tumors [179]. In an extensive analysis of 110 consecutive patients with a confirmed diagnosis of ACC between the years 1990 and 2017, PSMA expression was explored, and survival was analyzed using multivariate Cox-proportional hazard analysis; PSMA was found to be expressed in primary, recurrent, and metastatic ACC of both the salivary and seromucous glands [180]. In a case report of adenoid cystic carcinoma, PSMA PET/CT provided complementary staging and an initial treatment response assessment [181]. Because of the elevated level of PSMA expression in adenoid cystic carcinoma, ^68^Ga-PSMA PET/CT has a promising future as an imaging modality for this malignancy [182].

#### 2.1.7. Sinonasal Small-Round-Blue-Cell Tumors

The small-round-blue-cell tumors (SRBCT) constitute a heterogeneous group of malignant neoplasms characterized by a monomorphic population of undifferentiated cells with small-sized nuclei and scant cytoplasm [183].

In an undifferentiated tumor showing a small-round-blue-cell morphology, the series of tumors that must be considered in the differential diagnosis are melanoma, mesenchymal chondrosarcoma, rhabdomyosarcoma, SNUC, SCC (including NUT carcinoma), small-cell osteosarcoma, lymphoma, ONB, Ewing sarcoma/primitive neuroectodermal tumor, pituitary adenoma, and plasmacytoma [184].

In a study by Ozturk et al. [185], sinonasal SRBCT was shown to have numerous distinct imaging features on CT, MRI, and ^18^F-FDG PET/CT that could be useful in differentiating lesions when the histopathologic diagnosis is inconclusive. They also showed that a combination of ^18^F-FDG PET/CT and DWI increased the accuracy of sinonasal SRBCT characterization. While tissue sampling remains the key method for confirming the presence of tumors, relying solely on immunohistochemical analysis may not always provide conclusive results due to similarities between different types of tumors.

**Figure 7:**
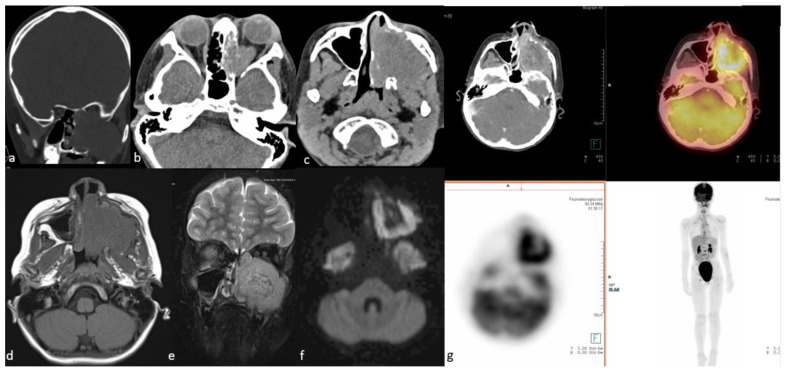
Pediatric sinonasal alveolar rhabdomyosarcoma. A 9-year-old female patient presenting with persistent left nasal drainage. Initial CT (**a**–**c**) shows an infiltrative soft tissue mass originating from the left maxillary sinus and nasal cavity with erosive changes in maxillary sinus walls and extension into the left masticator space and left extra/intraconal spaces. Subsequent MRI confirms the same findings (**d**–**f**) and shows increased signal on DWI (**f**), indicating high cellularity. The central portion of the lesion is necrotic. ^18^F-FDG PET/CT (**g**) again shows the central necrotic changes and, in addition, shows uptake in multiple lytic axial skeleton lesions. A biopsy revealed an alveolar rhabdomyosarcoma.

Rhabdomyosarcoma bears mentioning separately because of its important and distinct imaging features. They stand out with their highly aggressive behavior, such as adjacent tissue invasion, bone destruction, and intratumoral necrosis, compared to other SRBCTs [185] (Figure 7).

### 2.2. Benign Lesions

#### 2.2.1. Juvenile Nasopharyngeal Angiofibroma

Juvenile nasopharyngeal angiofibroma (JNA) is a rare benign fibrovascular, locally aggressive tumor exclusively seen in adolescent males [186]. In a series of five patients with JNA, all showed avid uptake of the SSTR analog ^68^Ga-dodecanetetraacetic 1-Nal3-octreotide (DOTANOC) [187]. Additionally, in postoperative follow-up, ^68^Ga-DOTANOC PET/CT appeared to be more specific than MRI in the identification of true residual/recurrent tumors [187]. In residual tumors, PET/CT with SSTR analogs may have an advantage over contrast-enhanced MRI in the diagnosis, decision-making, and planning of stereotactic radiation [187].

PSMA is expressed in the endothelial cells of the tumor-associated neovasculature of various non-prostatic benign and malignant neoplasms, including JNA. In a pilot study of 18 postoperative patients with suspicion of recurrence, ^68^Ga-PSMA-11 PET/CT was performed alongside conventional contrast-enhanced MRI. The sensitivity, specificity, and positive and negative predictive values of MRI were 100%, 33.33%, and 75% and 100%, respectively. The sensitivity, specificity, and positive and negative predictive values of ^68^Ga-PSMA-11 PET/CT were 100% for all parameters, suggesting it may play an important role in diagnosing, decision-making, planning radiation therapy, and surgical treatment [188].

#### 2.2.2. Inverted Papilloma

Inverted papilloma (IP) is an unusual benign epithelial neoplasm of the sinonasal cavity, accounting for 0.5% to 4.0% of all primary nasal neoplasms [189,190].

In a study of patients with suspected recurrence of IP, ^18^F-FDG PET/CT detected recurrence based on increased uptake in the lesion [191]. ^18^F-FDG PET/CT seems useful as a diagnostic tool for localization and determination of the extent of disease with the integration of both morphological and metabolic data [192].

^18^F-FDG PET/CT imaging has demonstrated high FDG uptake in IP with coexistent carcinoma [192].

#### 2.2.3. Sinonasal Schwannoma

Schwannomas are slow-growing and benign tumors thought to arise from Schwann cells, which are responsible for the myelination of nerve fibers. Roughly 25–45% of all schwannomas occur in the head and neck region, with the most affected location being the vestibulocochlear nerve [1]. Sinonasal schwannomas account for 4% of all head and neck schwannomas, with fewer than one hundred reported cases. The high uptake of ^18^F-FDG PET/CT in schwannomas is related to peritumoral lymphoid cuffs and glucose transporters [4]. Due to its ability to assess glucose metabolism intensity and tumor heterogeneity, ^18^F-FDG PET is widely used for the clinical differentiation of benign and malignant tumors. However, false-positive and false-negative ^18^F-FDG PET/CT results occur based on FDG uptake. The FDG uptake of benign schwannomas varies substantially, with occasional high uptake [193,194].

#### 2.2.4. Glomangiopericytoma

Sinonasal glomangiopericytoma is a rare mesenchymal neoplasm arising in the nasal cavity or paranasal sinuses [195,196]. It is considered a borderline low-malignant-potential soft tissue tumor [195,196,197,198].

Conrad et al. presented three cases of histologically proven glomangiopericytoma; there was relatively prominent uptake of Tc99m-methoxy isobutyl isonitrile (MIBI), ^18^F-FDG, and ^11^C-choline, for which the mechanism is not fully understood. Hypothetical explanations may include the high concentration of mitochondria in these tumors in the case of Tc99m-MIBI and the presence of neovasculature in glomangiopercytoma for all three radiopharmaceuticals [15].

Another case presented ^18^F-FDG PET/CT findings of glomangiopericytoma in a 53-year-old with nasal congestion and facial pressure symptoms. CT and MRI showed a nasal mass extending along the sphenoid ridge from the posterior nasal cavity into the posterior nasopharynx. PET showed the mass to have a uniformly low-grade FDG metabolism. Pathologic examination of the surgical specimen showed classic features of glomangiopericytoma [199]. A case study by Aras et al. also reported that PET/CT images showed marked hypermetabolism (SUVmax of 20.9) in the primary tumor, which was reported as a sinonasal-type hemangiopericytoma [200].

#### 2.2.5. Inflammatory Myofibroblastic Tumor

Inflammatory myofibroblastic tumor (IMT) is a rare tumor-like condition with characteristics of invasion, local recurrence, and chromosomal abnormality, although whether it is a tumor or reactive lesion is debatable [201]. Histologic diversity complicates diagnosis. This entity is most often seen in the lungs; however, it may also occur in the head and neck regions, with an incidence of 15% [202,203]. The most involved head and neck areas are the orbit, oral cavity, pharynx, larynx, and esophagus. The maxillary sinus is very rarely involved [204]. Bony destruction and invasion of adjacent sites are typical characteristics that may be misleading on imaging [205]. The CT and MRI findings are nonspecific and not much different from most other sinonasal malignancies. Moreover, the inflammatory nature of the tumor is associated with intense ^18^F-FDG uptake, a false positive for malignancy.

## 3. PET/CT Applications in Sinonasal Neoplasms Other Than ^18^F-FDG

### 3.1. Sinonasal Tumors of Neuroendocrine Differentiation

Sinonasal tumors with neuroendocrine differentiation are rare tumors. They are divided into two histologic groups of neuroectodermal and epithelial origin. The neuroectodermal group consists of olfactory neuroblastoma. The epithelial group consists of neuroendocrine tumors and neuroendocrine carcinomas [206].

#### 3.1.1. Olfactory Neuroblastoma (Esthesioneuroblastoma)

Olfactory neuroblastoma (ONB), also known as esthesioneuroblastoma, is a rare tumor arising from neuroectodermal cells in olfactory epithelium. It constitutes approximately 10% of sinonasal malignancies and 3–6% of intranasal malignant tumors [207,208].

The tumor starts insidiously in the upper parts of the nasal cavity, including the cribriform plate, the superior and middle turbinate, and the upper portion of the nasal septum, and can grow to involve the anterior cranial fossa, the nasal cavity, paranasal sinuses (especially ethmoids), and the orbit [209,210,211,212,213].

Regional metastases most commonly involve cervical lymph nodes, and it is well documented that cervical nodal metastasis is one of the most important prognostic factors for survival [214]. The most commonly involved cervical lymph nodes are level II lymph nodes; however, involvement of all cervical nodal levels, especially levels I–IV, and the retropharyngeal nodes has been reported [215]. Less commonly, metastasis to bone, lung, and liver may be seen, and this is also often delayed [210,211,212].

Local and loco-regional recurrence is common and can occur over a long period of time, necessitating lifelong follow-up. ONBs have a relatively good prognosis, especially when treated by surgery followed by radiotherapy.

The mainstay treatment of ONB usually consists of radical surgical resection [216]; however, combined modality therapy, including adjuvant radiotherapy, is beneficial in all stage groups, including the early stage [217]. Compared to surgery alone, postoperative radiotherapy results in more side effects but superior local control [218]. Institutional preference guides preoperative versus postoperative radiation for ONB, with both approaches demonstrating 5-year disease-free survival rates exceeding 85% [219,220]. Current National Comprehensive Cancer Network (NCCN) clinical guidelines recommend postoperative radiation therapy for all ONB patients. Observation can be considered in the setting of T1 N0 disease with clear surgical margins (R0) and without high-grade features, perineural invasion, or involvement of the cribriform plate or medial orbital wall [221].

Imaging plays a critical role in the diagnosis, staging, initial treatment decisions, and post-treatment management of ONB. CT and MRI are utilized to assess osseous involvement and soft tissue extension, respectively. ONBs are typically seen within or arising from the olfactory cleft, although ectopic lesions have been reported [222]. Calcifications within the tumor may also be detected by CT [223]. On MRI, ONBs classically have intermediate signal on T1-weighted images and mildly hyperintense signal on T2-weighted images. Homogeneous or heterogeneous enhancement can be seen on post-gadolinium images.

**Figure 8:**
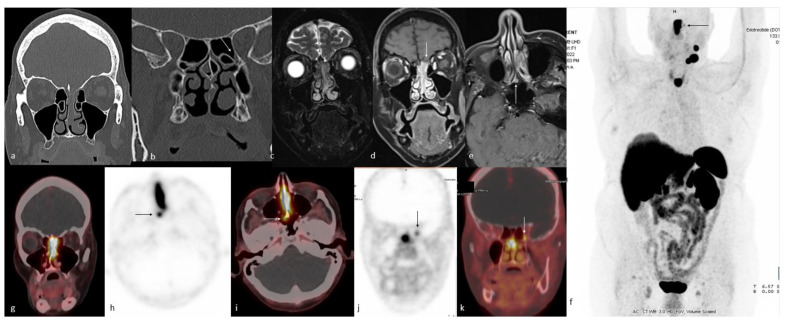
Olfactory neuroblastoma with metastases. A 71-year-old female presenting with a palpable left neck mass. CT (**a**,**b**) demonstrated a left nasal cavity mass with dehiscence of the cribriform plate on the left side. MRI images [coronal fat-saturated T2w (**c**), post-contrast fat-saturated T1w (**d**,**e**)] demonstrate a T2w isointense, enhancing left nasal cavity mass invading the olfactory fossa (arrow, (**d**)). The biopsy of the left nasal cavity mass was consistent with grade 2 olfactory neuroblastoma. Subsequently, ^68^Ga-DOTATOC PET/CT was performed. The MIP image (**f**) shows intense uptake in the left nasal cavity mass, with a small focus of uptake just laterally (arrow, (**f**)), and uptake in the left level IB and IIB lymph nodes and thyroid gland. Fused coronal (**g**) and axial (**i**) images show intense FDG uptake in the nasal cavity lesion with a small right paramidline focus of uptake at the level of the choanae (arrow (**h**,**i**)). Retrospectively, this can be identified as an enhancing focus on MRI (arrow, (**e**)). There is also another focus of uptake near the left orbital apex in the vicinity of the left posterior ethmoid air cells (arrow, (**j**,**k**)), which can be identified retrospectively as an area of mucosal thickening on initial CT (arrow, (**b**)).

Functional whole-body imaging with ^18^F-FDG PET/CT for detection of tumor glucose metabolism is a useful adjunct to conventional imaging for staging and surveillance following definitive therapy, as ONBs show high—though not universally so—metabolic activity [172,224,225]. Additionally, ONBs show high expression of somatostatin receptors (SSTR), which can be targeted by somatostatin analog imaging agents (Figure 8).

Ozturk et al. [51] analyzed ^18^F-FDG uptake in primary sinonasal neoplasms [51]. For ONB, SUVmax, SUVmean, and SUVratio-mediastinum were 8.7 ± 3.7 (±SD), 4.9 ± 21.7 (±SD), and 2.9 ± 1.3 (±SD), respectively. Broski et al. [225] showed in their retrospective study of ^18^F-FDG-PET/CT in ONB that both primary and metastatic diseases have high uptake (SUVmax of 8.7 ± 4.8 and 8.6 ± 6.5, respectively) and that ^18^F-FDG PET/CT is a helpful adjunct to conventional imaging in staging and restaging by detecting nodal and distant metastatic disease not demonstrated by conventional imaging and identifying local recurrence hidden by treatment changes on conventional imaging [225]. Wu et al. showed ^18^F-FDG uptake without variability in relation to ONB tumor size [172].

A study by Fujioka et al. [224] showed the usefulness of postoperative ^18^F-FDG PET/CT in the management of ONB. ^18^F-FDG PET/CT was able to distinguish local recurrence from inflammation when compared to MRI. Even for recurrent lesions that appear benign on MRI, ^18^F-FDG PET/CT can often be helpful for correctly diagnosing them as recurrent lesions. Another study by Broski et al. [225] demonstrated the usefulness of ^18^F-FDG PET/CT in the restaging of ONB by identifying local recurrence disguised by treatment changes on conventional imaging.

Studies show that ^18^F-FDG intensity within sinonasal tumors helps suggest underlying histology, but due to overlap in uptake intensity, biopsy continues to be necessary for a definitive diagnosis [169,172,225]. Elkhatib et al. [169] in a retrospective review of 13 patients (7 with ONBs and 6 with SNUCs) found a highly statistically significant difference in uptake between ONB and SNUC (mean SUVmax 7.2, range 4.6–10.7 versus mean SUVmax 35.6, range 10.8–77.9). However, other authors found a statistically significant difference in uptake in ONB compared with malignancies such as adenoid cystic carcinoma and sinonasal mucosal malignant melanoma [51].

ONBs show high expression of SSTR type 2 and variable expression of other somatostatin receptors. SSTR expression is a diagnostically important marker that can be imaged with somatostatin analog-labeled radionuclides, which have been described for ONB since the early 2000s with indium-111 (^111^In)-pentetreotide for planar and SPECT imaging and currently ^68^Ga-DOTATATE and ^68^Ga-DOTATOC for PET imaging [226,227]. Multiple case reports and case series have demonstrated SSTR-targeted imaging in ONB due to the greater specificity and higher target-to-background ratio with high tumor uptake in the absence of competing metabolic brain activity, which is problematic with ^18^F-FDG [95,227,228,229,230]. In one such case report, ^68^Ga-DOTATATE PET/CT was able to detect cervical node metastases in an ONB patient with suspicious sub-centimeter lymph nodes that were vague based on CT alone [11]. Moreover, another case report has shown the utility of ^68^Ga-DOTATATE PET/CT for the accurate staging of ONB in the sphenoclival region. ^68^Ga-DOTATATE PET/CT along with ^177^Lu-DOTATATE have been used in identifying SSTR-expressing ONB patients [231,232]. ^68^Ga-DOTATATE PET/CT has shown usefulness in the identification of residual tumors after resection and lesions in areas, including brain parenchyma, that did not show abnormal radiotracer uptake on ^18^F-FDG PET/CT scans. This finding is consistent with prior cases demonstrating that ^68^Ga-DOTATATE is superior to ^18^F-FDG PET/CT when it comes to the visualization of brain tissue [233,234]. Moreover, SSTR receptor expression is a theranostic target that allows for another therapeutic avenue. Sparse but intriguing data from case reports and a cohort study report successful administration of peptide receptor radionuclide therapy (PRRT) in patients with ONB [232,235]. ^68^Ga-DOTATATE PET/CT has been used to identify SSTR-expressing ONB patients for ^177^Lu-DOTATATE therapy [231,232]. Hasan et al. [232] reported a cohort of seven patients undergoing ^177^Lu-DOTATATE in unresectable recurrent or metastastic ONB and showed clinical and imaging responses in most patients. Theranostics offers a promising diagnostic and therapeutic option for these patients, though larger studies evaluating progression-free survival, overall survival, and quality of life in the setting of PRRT are needed [227].

#### 3.1.2. Sinonasal Neuroendocrine Tumor and Carcinoma

Neuroendocrine neoplasms (NENs) of epithelial origin consist of a spectrum of tumors ranging from slow-growing neuroendocrine tumors (NETs), including typical and atypical carcinoids, to highly aggressive neuroendocrine carcinomas (NECs), including small-cell and large-cell carcinomas of neuroendocrine type [236,237,238]. These tumors are rare, accounting for less than 5% of malignancies in the head and neck. They are most often found in the larynx, followed by the salivary glands and the sinonasal region [238,239].

Imaging is essential for staging. CT and MRI help with the demonstration of the degree of local invasion and cervical lymphadenopathy. PET/CT assesses regional and distant metastases. ^68^Ga-DOTATATE PET/CT is emerging as the superior imaging approach for detecting and evaluating patients with NETs. While SSTR-based imaging is the functional imaging of choice in diagnosing and managing NETs [14], in high-grade (Grade 2 and 3) NETs and NECs, ^18^F-FDG PET/CT is a better choice as these tumors lack or have lost SSTR expression [240]. A case series by Liu et al. demonstrated that ^68^Ga-DOTATATE imaging of the sinonasal region can improve the detection of unknown SSTR-expressing lesions and metastatic disease and enhance the accuracy of post-treatment restaging and tumor surveillance [229].

Survival is poor despite aggressive multimodal therapy [238]. Elective nodal irradiation is recommended for patients with moderate and poorly differentiated NEC [241].

## 4. PET/CT in the Follow-Up of Sinonasal Malignancies Treated with Radiotherapy

Radiotherapy has been commonly employed as a treatment strategy for sinonasal malignancies, and the delivery of enough radiation dose to the target is often difficult due to the critical organs in that area, including the eyes, optic nerves and chiasm, and brainstem. High-dose irradiation of these organs may cause toxicity, including permanent injury and late toxicity manifestations [242]. Sinonasal neoplasms have a high rate of recurrence following treatment, and clinicians utilize a variety of surveillance techniques. Local recurrence rates are generally estimated at 10–30%, even when surgical margins are pathologically negative [243,244,245].

In general, the guidelines for head and neck cancer as a broad category determine surveillance modality and follow-up frequency. However, recent studies [246,247,248,249] have demonstrated that a more tailored approach to follow-up may be necessary. De Visscher et al. [246] accentuated the impact of routine patient follow-up for head and neck cancer, demonstrating that survival improved significantly when asymptomatic recurrence was detected at routine visits. Alone, ^18^F-FDG PET/CT can identify 95% of asymptomatic recurrences in head and neck tumor patients in the first 24 months after completion of treatment, making it more sensitive than MRI and CT scans [247,248,249].

^18^FDG PET/CT has proven to be useful in recurrence detection in head and neck cancer, and it is recommended as an imaging modality following treatment by the NCCN guidelines [250]. Anzai et al. [251] used receiver operator characteristic analysis to compare ^18^F-FDG PET/CT and CT/MR imaging in patients with head and neck tumors and symptoms suggesting recurrence and no obvious tumor mass. ^18^F-FDG PET/CT demonstrated higher sensitivity and specificity, with a significant difference in the area under the curve. PET detected twice as many recurrences as physical examination and CT in routine surveillance of patients previously treated for squamous cell carcinoma of the head and neck [252].

A study by Ozturk et al. [92] suggested that surveillance ^18^F-FDG PET/CT should first be performed after 1 month following the conclusive treatment because, if performed earlier, the accumulation of ^18^F-FDG may represent treatment-induced inflammatory changes. Furthermore, small traces of recurrent tumors may not be seen on ^18^F-FDG PET/CT right after treatment but become visible a couple of weeks later. Other studies, including two systematic reviews with meta-analyses, have identified 10 to 12 weeks posttreatment as the ideal time-point for improved diagnostic accuracy of ^18^F-FDG PET/CT in so far as differentiating persistent viable disease from posttreatment inflammation for deep tissue sites of the head and neck [253,254,255].

A retrospective analysis undertaken by Schwartz et al. [256] suggested that ^18^F-FDG PET/CT typically demonstrates a prolonged period of hypermetabolism in deep tissue sites of the head and neck in patients previously treated for sinonasal malignancy. Their findings suggested reevaluating the previously described 10- to 12-week cutoff point for initial posttreatment ^18^F-FDG PET/CT for head and neck squamous cell carcinoma as applied to sinonasal malignancies.

## 5. Role of PET/CT in the Management of Sinonasal Malignancies Using Radiotracers Other Than FDG

^18^F-FDG is the most widely used PET radiotracer because the glucose metabolism of many malignant tumors causes them to readily take up the radiotracer that can be identified on PET/CT anywhere in the body in one session. Limitations of this radiotracer are its inaccuracy for lesions less than 1 cm because of the partial volume effect, low target-to-background ratio, difficulties in imaging non-FDG avid tumors, and motion artifact [3,9]. In addition, local high physiological FDG activity by tissues such as those of the brain and genitourinary tract can render the signal-to-noise ratio unfavorable for lesion detection and lead to false-negative results by masking malignant lesions [257]. Furthermore, ^18^F-FDG is not an excellent imaging modality for differentiating malignancies from postsurgical changes, post-radiation changes, and inflammatory processes. Such processes often lead to increased ^18^F-FDG activity and can lead to false-positive results [257].

For tumors expressing SSTR and with low ^18^F-FDG uptake, somatostatin analog imaging agents, such as ^68^Ga-DOTATOC/DOTATATE [10] or ^18^F-DOPA [98,258], can be used. Even though multiple ^68^Ga-labeled somatostatin analogs have been introduced to clinical practice, including ^68^Ga-DOTANOC, ^68^Ga-DOTATATE, and ^68^Ga-DOTATOC, ^68^Ga-DOTATATE was found to have the highest binding affinity for SSTR2 receptors [7]. ^68^Ga-DOTATATE PET/CT has a higher lesion-to-background contrast compared with the other imaging modalities, allowing for the detection of sub-centimeter abnormalities that cannot reliably be identified otherwise [14]. Studies have shown that ^68^Ga-DOTATATE PET/CT provides significant information that has a high impact on the management of patients with NETs, directing patients with identifiable primary tumor sites to curative surgery, patients with metastatic disease to systemic therapy, and optimizing management for patients [259,260,261]. Well-differentiated neuroendocrine tumors present with an elevated level of expression of SSTR and can, therefore, be studied with ^68^Ga-DOTA-peptides (^68^Ga-DOTANOC, ^68^Ga-DOTATOC, and ^68^Ga-DOTATATE) [262].

Since ONBs demonstrate high expression of SSTR-2 and variable expression of other somatostatin receptors, somatostatin analog-labeled radionuclides, such as ^68^Ga-DOTATOC and ^68^Ga-DOTATATE, are routinely used in routine clinical practice [226,227]. Recent literature proved that these radiotracers show superior specificity and target-to-background ratios with high tumor uptake in the absence of competing metabolic brain activity, which is a significant problem with ^18^F-FDG [95,227,228,229,230]. Another unique advantage of being able to detect SSTR receptor expression is that it can be used as a theranostic target and can guide the ^177^Lu-DOTATATE therapy in ONB patients [231,232].

There are several other radiotracers that are also gaining attention, such as F-DOPA. The DOPA radiotracer was shown to be effective for the diagnosis of several tumors, including carcinoid tumors, pheochromocytomas, insulinomas, and glomus tumors [98]. The scientific basis for imaging NETs with ^18^F-DOPA PET is the unique ability of these tumors to accumulate and decarboxylate L-DOPA in the catecholamine pathway [263]. In a study of 31 patients, more neuroendocrine tumors were detected using ^18^F-DOPA PET compared to when using both CT and MRI. The positive predictive value of the ^18^F-DOPA PET was estimated to be 92%, and its negative predictive value is 95% for NET detection [98]. ^18^F-DOPA PET is more accurate in the detection of well-differentiated neuroendocrine tumors than poorly differentiated ones. In a study involving 25 patients with histologically proven NET, patient-based sensitivities were 96% for ^68^Ga-DOTATATE PET and 56% for ^18^F-DOPA PET. ^68^Ga-DOTATATE PET showed metastases in 54 of the 55 positive metastatic tumor regions, whereas ^18^F-DOPA PET showed metastases in only 29 of the 55 tumor regions [264].

One promising imaging modality is ^68^Ga-FAPI-46. In a single-center exploratory study comparing the performance of ^68^Ga-FAPI-46 with the standard ^18^F-FDG PET/CT, ^68^Ga-FAPI-46 PET/CT showed a comparable diagnostic performance to ^18^F-FDG PET/CT for both the initial staging and recurrence detection in head and neck SCC patients [99]. Biodistribution of ^68^Ga-FAPI is low as compared to other tracers, including ^18^F-FDG, improving the detection of lesions at the brain, liver, head and neck, peritoneum, mesentery, omen tum, and axial skeleton level [262]. In one case report, ^68^Ga-FAPI-46 helped in the diagnosis of malignant melanoma of the nasal cavity [265]. In another case report, ^68^Ga-FAPI-46 was used in the diagnosis of chondrosarcoma of the nasal cavity [266].

Recent articles showed that PSMA was found to be expressed by primary, recurrent, and metastatic adenoid cystic carcinoma of salivary and seromucous glands, and ^68^Ga-PSMA PET/CT has a promising future for the management of these tumors [180,181,182].

Another imaging technique is ^11^C-choline PET/CT [267]. ^11^C-choline, a precursor in the phosphatidyl-choline biosynthesis pathway, has also been shown to be a useful marker for the detection of malignant tumors with an elevated concentration of phosphatidylcholine in the cell membrane. The main utility of ^11^C-choline PET/CT is to provide higher-contrast images of head and neck tumors as compared to those provided by ^18^F-FDG PET/CT [98]. Unfortunately, little is known about the utility of choline in sinonasal malignancies [267]. Compared to ^18^F-FDG PET/CT, ^11^C-choline PET/CT has not been shown to be better for the detection of recurrences of head and neck tumors after combined therapy except for tumor recurrences at or close to the skull base, mainly because of the confounding uptake of FDG in the adjacent brain [16].

Other radiotracers, such as ^11^C-methionine, have also been studied. The utility of ^11^C-methionine has already been documented in several types of malignancies, such as brain neoplasms, breast cancer, and tumors of the head and neck region [268,269,270]. In addition, other less common radiotracers, such as ^64^Cu-MeCOSar-Tyr^3^-octreotate (SARTATE) and ^68^Ga-CXCR4, may provide both new diagnostic and therapeutic options for NEN patients [262].

Finally, one can predict that either of these radiotracers (^11^C-choline, ^11^C-methionine, or FAPI) can have potentially significant roles in the management of sinonasal malignancies in the future.

The added clinical impact of PET/CT imaging with various radiotracers has been shown in Table 1.

## 6. Conclusions

Sinonasal cancers are a rare group of malignancies with a mostly unfavorable prognosis. Surveillance imaging has a crucial role in management due to their high rate of recurrence despite optimal treatment and presentation at an advanced stage. In addition to conventional imaging modalities, such as US, CT, and MRI, ^18^F-FDG PET/CT has very high sensitivity for the diagnosis and characterization of sinonasal malignancies. It provides functional information along with morphology obtained by CT and MRI throughout the entire body in one session. ^18^F-FDG PET/CT not only helps with the accurate staging of the primary disease at presentation, which is highly predictive in the determination of survival, but also has an essential role in detecting local, regional, or distant recurrences. ^18^F-FDG PET/CT is a reliable modality for determining the treatment response to both chemotherapy and/or radiotherapy in sinonasal malignancies. SSTR analog PET agents and ^11^C-choline were also very helpful in treatment planning, surveillance of some histologic subtypes of sinonasal malignancies, and determining the treatment response.

Almost all the classical limitations of PET/CT also apply to sinonasal tumor imaging. The most well-known of these is the difficulty in the identification of brain metastases due to the high background metabolism and the differentiation of small metastases where the spatial resolution of PET/CT is not enough [144]. Several other problems can make the PET interpretation challenging, such as variable physiologic uptake of the radiotracers by normal tissues, radiotracer uptake due to non-neoplastic processes such as inflammation or infection, occasional malignant lesions with low avidity (mostly for FDG), unusual tumor sites, misregistration, and motion artifacts [271].

The SPECT/CT provides several new developments, such as quantification of radiopharmaceutical uptake in absolute units using cadmium zinc telluride detectors and novel non-parallel hole collimators, and the ability to obtain whole-body SPECT acquisitions with multiple bed positions. These are recently implemented in routine clinical practice and result in a high-quality attenuation correction and co-registration/fusion of the metabolic, and anatomical images [272]. Another important development in this regard is undoubtedly the long axial field-of-view PET/CT scanners, which overcome the limitations of the standard axial field-of-view scanners with suboptimal signal-to-noise ratio and image quality [273]. These new techniques will also improve whole-body cancer imaging, including the head and neck region.

## Figures and Tables

**Table 1:** The added value of PET/CT imaging with various radiotracers in common sinonasal malignancies.

Tumor Type	Primary Radiotracer	Added Value of PET/CT to Diagnosis
Adenocarcinoma	^18^F-FDG	- Important in the follow-up and detection of the recurrence.
Squamous Cell Carcinoma	^18^F-FDG	- SUVmax generally shows aggressiveness, but is also associated with favorable prognosis due to better response to treatment; - Important in the follow-up and detection of the recurrence.
Undifferentiated Carcinoma	^18^F-FDG	- Important in the follow-up and detection of the recurrence.
Malignant Melanoma	^18^F-FDG	- Important in the follow-up and detection of the recurrence.
Lymphoma	^18^F-FDG	- Crucial for determining the proper biopsy site. - Important in the follow-up and detection of the recurrence.
Adenoid Cystic Carcinoma	PSMA ^18^F-FDG	- Important in the follow-up and detection of the recurrence.
Neuroendocrine Tumor	Somatostatin Analogs	- Somatostatin receptor-based imaging is the imaging of choice in diagnosis and management; - ^68^Ga-DOTATATE has the highest binding affinity for SSTR2 receptors of the neuroendocrine tumors.
Esthesioneuroblastoma(Olfactory Neuroblastoma)	Somatostatin Analogs ^18^F-FDG	- Relatively lower SUVmax and SUVmean compared to common sinonasal cancers; - If the DOTATATE receptor is positive, 177-Lu DOTATATE (Lutathera^®^) can be used in treatment.

DOTATATE: dodecanetetraacetic acid-Tyr3-octreotate; FDG: fluorodeoxyglucose; SUV: standard uptake value.

## Abbreviations

^18^F-FDG PET/CT	18-Florine-fluorodeoxyglucose positron emission tomography integrated with computed tomography
^68^Ga	68-Galium
ACC	Adenoid cystic carcinoma
ADC	Apparent diffusion coefficient
AIDS	Acquired immunodeficiency syndrome
DLBCL	Diffuse large B-cell lymphoma
DOPA	*Dihydroxyphenylalanine*
DOTATATE	Dodecanetetraacetic acid-Tyr3-octreotate
DOTATOC	Dodecanetetraacetic acid-D-Phe1-Tyr3-octreotate
DOTANOC	Dodecanetetraacetic 1-Nal3-octreotide
DTPAOC	Diethylenetriaminepentaacetic acid-d-phenylalanine-octreotide
DWI	Diffusion-weighted imaging
EBV	Epstein–Barr virus
F	Florine
FAPI	Fibroblast activation protein
FDA	Food and Drug Administration
FDG	Fluorodeoxyglucose
FLT	Fluorothymidine
HIV	Human immunodeficiency virus
HPV	Human papillomavirus
IACRT	Intra-arterial chemoradiotherapy
IMRT	Intensity-modulated radiation therapy
IMT	Inflammatory myofibroblastic tumor
IP	Inverted papilloma
MIBI	Methoxy isobutyl isonitrile
MRI	Magnetic resonance imaging
NEC	Neuroendocrine carcinoma
NEN	Neuroendocrine neoplasm
NET	Neuroendocrine tumor
NHL	Non-Hodgkin lymphoma
NK	Natural killer
NUT Carcinoma	Nuclear protein in testis carcinoma
ONB	Olfactory neuroblastoma also known as esthesioneuroblastoma
PNTS	Perineural tumor spread
PET	Positron emission tomography
PSMA	Prostate-specific membrane antigen
SARTATE	MeCOSar-Tyr^3^-octreotate
SCC	Squamous cell carcinoma
SD	Standard deviation
SIADH	Syndrome of inappropriate antidiuretic hormone secretion
SMMM	Sinonasal mucosal malignant melanoma
SNUC	Sinonasal undifferentiated carcinoma
SPECT	Single-photon emission computed tomography
SRBCT	Small-round-blue-cell tumors
SSTR	Somatostatin receptor
SUV	Standard uptake value
SUVmax	Maximum standard uptake value
SUVmean	Mean standard uptake value
T1w	T1-weighted
T2w	T2-weighted
WHO	World Health Organization
